# The use of dielectric blood coagulometry in the evaluation of coagulability in patients with peripheral arterial disease

**DOI:** 10.1186/s12907-017-0054-z

**Published:** 2017-08-23

**Authors:** Kimihiro Igari, Toshifumi Kudo, Takahiro Toyofuku, Yoshinori Inoue

**Affiliations:** Division of Vascular and Endovascular Surgery, Department of Surgery, Tokyo Medical and Dental University, 1-5-45, Yushima, Bunkyo-ku, Tokyo, 113-8519 Japan

**Keywords:** Peripheral arterial disease, Dielectric blood coagulometry, Blood coagulation, Diabetes mellitus, Activated partial thromboplastin time

## Abstract

**Background:**

Platelets and coagulation proteins contribute to the development of peripheral arterial disease, especially atherosclerotic disease. Several experimental studies have proven a significant correlation between hypercoagulability and atherosclerosis. We used dielectric blood coagulometry, which was initially designed to evaluate the coagulable status, to examine the coagulability of peripheral arterial disease patients, and investigated the factors that were significantly correlated with the results.

**Methods:**

We performed dielectric blood coagulometry in 49 peripheral arterial disease patients. In addition, we recorded the patients’ demographic information, including the presence of comorbidities, hemodynamic status, and laboratory findings. To investigate coagulability, we calculated the T_max_ value, which indicates the time from recalcification to maximum normalized permittivity.

**Results:**

The T_max_ values of diabetes mellitus patients were significantly lower than those of non-diabetic patients (1 MHz, *P* = 0.010; 10 MHz, 0.011). Furthermore, the T_max_ value was statistically correlated with the activated partial thromboplastin time (1 MHz, ρ = 0.286, *P* = 0.048; 10 MHz, ρ = 0.301, *P* = 0.037).

**Conclusions:**

Dielectric blood coagulometry detected the hypercoagulable status in diabetes mellitus patients, and reflected their level of coagulability, which was also evaluated by the activated partial thromboplastin time.

## Background

Peripheral arterial disease (PAD), especially atherosclerotic obliterans, is usually characterized by chronic inflammatory disease [1]. The inflammatory reaction, which is affected by local inflammation due to leukocytes, monocytes and macrophages, leads to endothelial dysfunction [2]. Although platelets have been shown to contribute to the development of atherosclerotic disease [3], the potential role of coagulation proteins remains to be proven. However, several experimental studies have shown a significant association between hypercoagulability and the development of atherosclerosis [4].

A number of measures are used to evaluate coagulability, including the prothrombin time, the international normalized ratio (PT-INR), and the activated partial thromboplastin time (APTT). Even though these tests might evaluate the bleeding tendency, they do not properly reflect the actual hypercoagulable status in vivo [5]. Furthermore, several enzymatic factors in the plasma components and blood cells have shown to affect hypercoagulability, which are not taken into account in the PT-INR and APTT tests [6].

Dielectric blood coagulometry (DBCM), is a recently developed method of evaluating coagulability [7, 8]. DBCM calculates the temporal changes in whole blood dielectric permittivity, and evaluates the coagulable status. Hasegawa et al. [9] reported the application of DBCM in the evaluation of hypercoagulability in patients with several risk factors for atherosclerosis; however, no studies have used DBCM to investigate coagulability in PAD patients.

In the present study, we conducted the DBCM test for the patients with PAD. Furthermore, we investigated the relationships between the DBCM results and various parameters.

## Methods

### Patient selection

Between September 2014 and June 2015, 49 patients with PAD due to atherosclerosis who were treated at the outpatient clinic of Tokyo Medical and Dental University Hospital were recruited for the present study. All of the patients provided their written informed consent, then we enrolled. This study was approved by the ethics committee of Tokyo Medical and Dental University (No. 701).

PAD was diagnosed based on the presence of >50% vessel stenosis due to lesions in the lower limbs. We mainly assessed the vessel stenosis by computed tomography angiography. In the cases with contraindication of using contrast media, such as chronic kidney disease and allergy, we evaluated the stenosis using magnetic resonance angiography and/or duplex ultrasound sonography. In this study, PAD of all patients was due to the atherosclerosis. Furthermore, in the present study, we included patients who previously underwent revascularization procedures to treat PAD lesions. We excluded the patients who had a history of recent malignant disease, systemic inflammatory disease, treatment with anticoagulants, or abnormal bleeding.

We retrospectively obtained the patients’ demographics, medications, and medical histories using a dedicated database. The patients’ medical records were reviewed as described below. Hypertension was defined as a systolic blood pressure of >130 mmHg, a diastolic blood pressure of >80 mmHg, or a history of treatment for hypertension. Dyslipidemia was defined as a serum low-density lipoprotein cholesterol level of >140 mg/dl, a high-density lipoprotein cholesterol level of <40 mg/dl, a triglyceride level of >150 mg/dl, or a history of treatment for dyslipidemia. Coronary arterial disease (CAD) was defined as the presence of angina pectoris, myocardial infarction or both, as documented on coronary angiography or based on a history of having undergone any coronary artery revascularization procedures. Cerebrovascular disease (CVD) was defined as a history of stroke, transient ischemic attacks, carotid artery revascularization, or cerebral hemorrhage. Chronic kidney disease (CKD) was defined as an estimated glomerular filtration rate of <60 ml/min/1.73 m^2^, which was calculated by the serum creatinine level, age, and gender. Diabetes mellitus (DM) was defined as a fasting blood glucose level of >126 mg/dl, a hemoglobin A1c level of >6.5%, or the use of antidiabetes medication. The severity of PAD was assessed by the measurement of the ankle brachial pressure index (ABI), which was calculated as the ankle systolic blood pressure divided by the brachial systolic blood pressure using a VasoGuard P84™ system (SciMed Ltd., Bristol, UK). Furthermore, the clinical severity of PAD was assessed by the Rutherford classification [10].

### The measurement of the collected blood samples

After the patients had fasted for at least 12 h, we collected blood samples by venipuncture. The samples were kept in tubes containing 3.13% sodium citrate. Complete blood cell counts, biochemistry examinations, and coagulation tests were conducted via standard laboratory methods in our hospital. The blood samples that were used for DBCM were kept at room temperature and were examined at 3–5 h after collection.

### Dielectric blood coagulometry

**Fig. 1:**
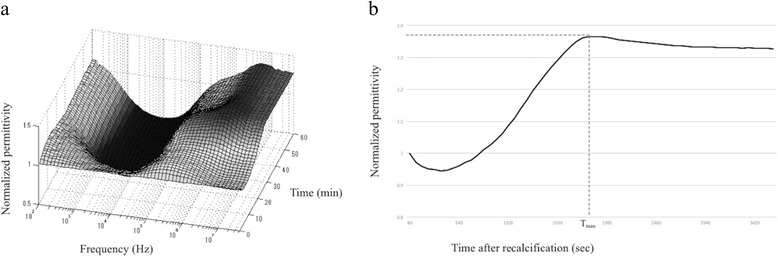
Temporal changes in the dielectric permittivity. **a** The normalized permittivity after recalcification is plotted according to the time and frequency. **b** A normalized permittivityat 1 MHz or 10 MHz is plotted, and calculates Tmax. *Tmax*, the time from recalcification to maximum normalized permittivity

We performed DBCM using a prototype dielectric coagulometer (Sony Corp., Tokyo, Japan) in accordance with the methods of previous studies [7, 8, 11]. In summary, we kept the blood samples at 37 °C, and DBCM was completed at 60 min after recalcification. The measurement frequency at which DBCM measured the dielectric permittivity was ranged from 100 Hz to 10 MHz, the sampling intervals were 1 min. DBCM was performed using a 180-μl citrated whole blood sample, and blood coagulability was assessed by recalcification using 15 μl of 308 mM CaCl_2_. A typical DBCM result is shown in a 3D plot of permittivity against the time and frequency in Fig. 1a. Hayashi et al. [8] reported that the time of dielectric coagulation obtained from the time-dependent permittivity has been well correlated with the coagulation time which was evaluated by the rheologic measurement. Then, coagulability was evaluated as the normalized permittivity according to the time series and frequency. Hayashi et al. [7] reported that the monitoring of the dielectric response at 1 MHz made it possible to obtain the clotting time, while Hasegawa et al. [9] evaluated the temporal change in the dielectric permittivity at 10 MHz, and showed a significant correlation with a hypercoagulable status. We therefore measured the change of permittivity at both 1 and 10 MHz, and evaluated the coagulability. DBCM showed a gradual increase in dielectric permittivity, which indicated the temporal acceleration of the coagulation. We therefore defined “T_max_,” which represents the time from recalcification to maximum normalized permittivity, as a parameter of coagulability (Fig. 1b). The repuroducibility of this DBCM test has been already evaluated by previous study [9].

### Statistical analysis

The continuous variables are expressed as the median and interquartile range (IQR), and the categorical variables are expressed as the frequency and percentage. Statistical significance was assessed using the Mann-Whitney *U* test for comparisons between two groups. Correlation was assessed using Spearman’s rank correlation coefficient, which reflects the degree of correlation between variables. *P* values <0.05 were considered to indicate statistical significance. The statistical analyses were performed using the Stat View software program (version 5, Abacus Concept Inc., Berkley, CA, USA).

## Results

### Patient demographics

We evaluated 49 (41 males and 8 females) PAD patients in the present study. The median age was 70 years (IQR, 63–77), and the median body mass index was 22.7 kg/m^2^ (IQR, 20.8–25.1). The documented comorbidities included smoking history (85.7%), hypertension (65.3%), dyslipidemia (51.0%), CKD (22.4%), CVD (18.4%), and CAD (10.2%). All patients with DM (44.9%) were diagnosed due to the medication of antidiabetes. The patient’s medications included Ca-blockers, *n* = 24 (49.0%); β-blockers, *n* = 6 (12.2%); angiotensin converting enzyme inhibitors, *n* = 2 (4.1%); angiotensin II receptor blockers, *n* = 20 (40.8%); statins, *n* = 22 (44.9%); and antiplatelet drugs, *n* = 44 (89.8%). None of the patients took anticoagulant drugs. By the Rutherford classification, 21 patients were classified as category 1, 14 patients as category 2, 8 patients as category 3, and 6 patients were divided in category 4.

### Comparisons according to the patient demographics

We performed DBCM and calculated the T_max_ at 1 MHz and 10 MHz. The median T_max_ values at 1 MHz and 10 MHz were 1320 s (IQR, 1140–1620 s) and 1560 s (IQR, 1380–1920 s), respectively. We assessed the DBCM tests according to the patients’ comorbidities and demographics (Table 1). The patients with DM showed significantly shorter T_max_ values than those without DM (*P* = 0.010 at 1 MHz, and *P* = 0.011 at 10 MHz, respectively). Interestingly, the T_max_ values of the patients who were receiving antiplatelet therapy did not differ to a statistically significant extent from those of the patients who did not receive antiplatelet therapy.

**Table 1:** Patient demographics and comparisons

		1 MHz, Tmax		10 MHz, Tmax	
Variables	Number	Median	*P*-value	Median	*P*-value
Gender (Male: Female)	41: 8	1320: 1260	0.569	1620: 1500	0.705
Smoking history (+: -)	42: 7	1320: 1320	0.841	1560: 1800	0.852
Hypertension (+: -)	32: 17	1290: 1440	0.338	1500: 1740	0.152
Dyslipidemia (+: -)	25: 24	1260: 1440	0.173	1500: 1650	0.652
Coronary artery disease (+: -)	5: 44	1320: 1320	0.987	1740: 1560	0.741
Cerebrovascular disease (+: -)	9: 40	1200: 1410	0.1473	1500: 1650	0.224
Chronic kidney disease (+: -)	11: 38	1080: 1380	0.167	1260: 1710	0.052
Diabetes mellitus (+: -)	22: 27	1170: 1500	0.010	1470: 1800	0.011
Ca-blocker (+: -)	24: 25	1320: 1320	0.711	1500: 1680	0.331
β-blocker (+: -)	6: 43	1500: 1320	0.492	1680: 1560	0.783
ACE-I (+: -)	2: 47	1440: 1320	0.667	1620: 1560	0.859
ARB (+: -)	20: 29	1290: 1320	0.714	1500: 1680	0.154
Statin (+: -)	22: 27	1260: 1440	0.487	1620: 1560	0.840
Antiplatelet (+: -)	44: 5	1350: 1320	0.620	1560: 1620	0.408

*IQR* interquartile range; *ACE-I* angiotensin converting enzyme – inhibitor; *ARB* angiotensin II receptor blocker

### The correlations between T_max_ and the other parameters

The correlations between T_max_ and each of the parameters are shown in Table 2. With the exception of the APTT (ρ = 0.286, *P* = 0.048 at 1 MHz, and ρ = 0.301, *P* = 0.037 at 10 MHz, respectively), none of the factors was significantly correlated with the T_max_ value.

**Table 2:** Correlations with several parameters

		1 MHz, Tmax	10 MHz, Tmax
Variables	Median, [IQR]	ρ, *P*-value	ρ, *P*-value
Age (years)	70, [63-77]	−0.219, 0.129	−0.219, 0.130
BMI (kg/m^2^)	22.7, [20.8-25.1]	0.080, 0.577	0.035, 0.807
ABI	0.95, [0.78-1.07]	0.019, 0.895	0.036, 0.803
White blood cell (/μl)	6400, [5100-7600]	−0.184, 0.202	−0.162, 0.262
Hemoglobin (g/dl)	13.9, [12.9-15.1]	0.203, 0.159	0.255, 0.078
Platelet (×10^4^/μl)	22.7, [20.2-26.9]	−0.126, 0.382	−0.060, 0.678
PT-INR	0.98, [0.94-1.01]	0.223, 0.122	0.125, 0.385
APTT (sec)	28.9, [27.5-31.6]	0.286, 0.048	0.301, 0.037
Fibrinogen (mg/dl)	302, [269-339]	−0.207, 0.151	−0.058, 0.688
Albumin (g/dl))	4.1, [3.8-4.4]	0.213, 0.189	0.212, 0.192
Creatinine (mg/dl)	0.89, [0.75-1.0]	−0.104, 0.472	−0.107, 0.457
Total cholesterol (mg/dl)	185, [166.3-207.8]	−0.057, 0.697	0.048, 0.741
Triglycerides (mg/dl)	118, [80.5-165]	0.061, 0.681	0.100, 0.500
LDL (mg/dl)	106, [94.5-121.8]	0.195, 0.224	0.182, 0.256

*BMI* body mass index; *ABI* ankle brachial pressure index; *APTT* activated partial thromboplastin time; *LDL* low-density lipoprotein; *IQR* interquartile range; *PT-INR* Prothrombin time – International normalized ratio

## Discussion

A comprehensive test that can evaluate the coagulable status is essential for the effective control of coagulability. DBCM might meet these necessities by measuring the changes in the permittivity [7]. In the present study, we evaluated the changes in permittivity at 1 MHz and 10 MHz. The dielectric permittivity of blood mainly changes at frequencies that range from hundreds of kilohertz to 10 MHz. The change occurs due to the accumulation of charge at the interface between the cytoplasm and the erythrocyte membrane [12]. Hayashi et al. [11] reported that the temporal changes in permittivity at 1 MHz reflected the clotting time. On the other hand, Hasegawa et al. [9] reported that DBCM represented a gradual increase in the dielectric permittivity from 2.5–16 MHz, and they focused on the temporal change in the dielectric permittivity at 10 MHz. Thus, both 1 and 10 MHz might be reasonable frequencies for evaluating coagulability based on the temporal changes in the permittivity. Moreover, Hayashi et al. [7] reported that the T_max_ parameter, which was referred to as “T_i(DS)_” in their report, was useful for evaluating the coagulability. In our study, we evaluated the temporal changes in permittivity at frequency of 1 and 10 MHz, and calculated the ‘T_max_’ value. We showed the significant correlation with the T_max_ and APTT, which represented the coagulable status, and DBCM might indicate the coagulability.

Our study showed a statistically significant correlation between the T_max_ and APTT values. Shortened APTTs are generally considered to be laboratory artifacts that arise from problematic venipuncture. However, there is increasing evidence to support that shortened APTT values may in some cases reflect a hypercoagulable state, which is potentially associated with an increased risk of thromboembolism [13, 14]. Tripodi et al. [15] reported that hypercoagulability, as detected by a shortened APTT value, was significantly associated with the occurrence of venous thromboembolism (VTE). In line with these reports, Hayashi et al. [8] found that DBCM allows quantitative monitoring of blood coagulability and that it is promising technique for the evaluation of VTE. Even though this mechanism has been uncertain, Hasegawa et al. [9] reported that the APTT was positively correlated with the end of acceleration time, which is almost the same as the T_max_. The APTT might reflect the intrinsic pathways of coagulation cascade [16]. Therefore, the T_max_ might also be affected by the intrinsic pathways, and potentially be used to assess the coagulable status as same as the APTT.

Patients with DM showed significantly shorter T_max_ values at 1 and 10 MHz than those without DM in the present study. This is because hyperglycemia contributes to the hyperfibrinogemia and activates the coagulative cascade, leading to an increase in thrombin formation and in the levels of fibrinogen degradation products [17]. However, the exact mechanisms of the development of the hypercoagulable state in diabetic patients is likely to multifactorial and is not yet completely understood. Lippi et al. [18] reported that the APTT values were significantly shortened in DM patients. Similarly, Zhao et al. [19] found that the DM patients showed statistically shortened APTT values in comparison to patients without DM. Similarly, we revealed that DM patients showed significantly shortened APTT values (median, 28.5 s) in comparison to patients without DM (29.4 s) (*P* < 0.001). Thus, the present study shows that the T_max_ values yielded by DBCM are significantly correlated with both the coagulable state (as reflected by the APTT) and a hyperglycemic status, which leads to hypercoagulability.

There were no significant differences in the T_max_ values of patients who were treated with/without antiplatelet drugs. The same result was shown in a previous report [9]. In addition to DBCM, several modalities, including thromboelastrography and rotational thromboelastometry, can be used to assess whole blood coagulability. These modalities can be used to evaluate the effects of antiplatelet treatment [20]. Even though we theoretically evaluated the effects of antiplatelet drugs, which led to an increased T_max_ value, we did not observe any differences. This might be due to the small sample size, which was one of the limitations associated with the present study. Both the APTT value and the presence of DM might have affected the association between the T_max_ and the use of antiplatelet drugs. Although the platelet counts of diabetic patients are normal, multiple studies have shown evidence of enhanced activation or increased platelet activity in DM patients [21]. Our study included 15 patients who received sarpogrelate hydrochloride as antiplatelet therapy. The drug, which is a selective 5-hydroxytryptaminen 2A receptor antagonist that is used in the treatment of diabetic patients with PAD, suppresses platelet aggregation [22]. It was very interesting that antiplatelet drugs, including aspirin, clopidogrel, and cilostazol did not significantly affect the patients’ T_max_ values, but that patients who were treated with sarpogrelate hydrochloride showed increased T_max_ values at 1 and 10 MHz in comparison to patients who did not receive the drug (1 MHz: median T_max_, 1500 vs. 1260, *P* = 0.044; 10 MHz: 1860 vs. 1500, *P* = 0.027). These discrepancies might be due to the small number of subjects and/or a bias associated with the inclusion criteria. Future studies in a larger population might reveal the effects of antiplatelet drug by DBCM.

## Conclusions

We herein demonstrated that the measurement of the T_max_ value by DBCM detected a change in the coagulable status according to the APTT value and the presence of DM. Even though the use of DBCM did not reveal the exact effects of antiplatelet drugs in the present study, the T_max_ values of patients who received sarpogrelate hydrochloride, an antiplatelet drug, were increased in comparison to those of patients who did not receive the drug. A more sensitive measurement with DBCM might therefore be successful in elucidating the effects of antiplatelet drugs.

## Abbreviations

ABI: Ankle brachial pressure index
APTT: Activated partial thromboplastin time
CAD: Coronary arterial disease
CKD: Chronic kidney disease
CVD: Cerebrovascular disease
DBCM: Dielectric blood coagulometry
DM: Diabetes mellitus
IQR: Interquartile range
PAD: Peripheral arterial disease
PT-INR: Prothrombin time, the international normalized ratio
VTE: Venous thromboembolism
